# Supplementary material for: Factors associated with in-hospital mortality of adult tetanus patients–a multicenter study from Bangladesh
Source: PLoS Negl Trop Dis. 2022 Mar 1;16(3):e0010235. doi: 10.1371/journal.pntd.0010235 (PMC8887756; doi:10.1371/journal.pntd.0010235)
Supplement: S1 Table — (DOCX) [file pntd.0010235.s001.docx]

**S1 Table. Questionnaire**

SL No: ................... Date of interview…………………. Date of admission……………………..

**Section A: Socio-demographic information**

| **SL No** | **Questions** | **Response** | **Code** |
| --- | --- | --- | --- |
| 1.1 | How old are you? (in complete years) | ................ years |  |
| 1.2 | What is your religion? | a) Islam =1, b) Hinduism =2,  c)Christian = 3, d) Buddhism =4,  e)Other =99 specify…………………….. |  |
| 1.3 | What is your educational status? | a) Illiterate =1,  b) Primary = 2 (up to 5 class),  c) Secondary =3 (up to 10 class),  d) Higher secondary = 4 (up to 12 class),  e) Graduate =5,  f) Above graduate=6  Others =99 (please specify...........................) |  |
| 1.4 | What is your marital status? | a) Single =1, b) Married =2, c) Widow =3, d) Divorced =4 |  |
| 1.5 | What is your occupation? | a) Housewife =1, b) Teacher =2,  c) Banker=3, d) NGO workers =4,  e) Businessman=5, f) Service Holder= 6,  g) Farmer = 7, h) Others =99 (please specify)  ......................................................... |  |
| 1.6 | What is your monthly household income? | 1= <7500 taka  2=7501-10,000 taka  3=10,001-15000 taka  4=>15000 taka |  |
| 1.7 | Place of residence | 1=Urban  2=Rural |  |
| 1.8 | Smoking history | 1. Current smoker 2. Past smoker 3. Never smoker |  |

**Section B: Information regarding tetanus**

| 2.1 | Reasons for tetanus: | 1=Post-traumatic  2=Post-surgery |  |
| --- | --- | --- | --- |
| 2.2 | Place of wound: | 1=Head or face  2=Extremities  3=Trunk |  |
| 2.3 | Period of onset (Duration between onset of symptoms and hospital admission): | In days: …………….  1= <3 day  2= ≥3 day |  |
| 2.4 | Incubation period (Duration between causative injury and symptom onset): | In days: ……………  1= <7 day  2= ≥ 7 day |  |
| 2.5 | Clinical features: | 1=Trismus (lock jaw)- No/Mild/Moderate/Severe  2=Risus sardonicus - No/Yes  3=Dysphagia – No/Mild/Severe  4=Spasms – Short/Prolonged  5=Spasticity – No/Yes  5=Neck stiffness – No/Yes  6=Rigidity - Localized/generalized  7= Abdominal rigidity – No/Yes  8=Hyperreflexia – No/Yes  9=Hypertonia – No/Yes  10=Opisthotonus – No/Yes  11=Fever/pyrexia – No/Yes  12= Apneic spells – No/Yes  13= Palpitation – No/Yes  14= Urinary retention – No/Yes  12=Others………….. |  |
| 2.6 | Examination | GCS: E - /4 , V- /5 , M- /6  **Pulse –**  Blood pressure -  Temperature –  **Respiratory rate -** |  |
| 2.7 | Patient had history of receiving tetanus vaccine: | 1=Yes  2=No  3= Don’t know |  |
| 2.8 | Tetanus vaccine after trauma: | 1=Yes  2=No  3=Don’t know |  |
| 2.9 | Tetanus Severity (Ablett): | 1=Mild  2=Moderate  3=Severe  4=Very severe |  |
| 2.10 | Types of tetanus: | 1=Generalized  2=Local  3=Cephalic (Opthalmoplegia/Supranuclear oculomotor palsy/Horner syndrome) |  |

**Section C: Comorbidities, investigation and treatment**

| 3.1 | Co-morbid diseases | 1=Diabetes mellitus  2=Chronic renal failure  3=COPD  4=Hypertension  5=Stroke  6=Ischemic heart disease  7=Malignancy  8=Others |  |
| --- | --- | --- | --- |
| 3.2 | Investigations |  |  |
|  | Complete blood count  Random Blood Sugar ………………….  Serum calcium …………………………  Serum creatinine……………………….  Serum electrolyte………………………  Others | Hemoglobin-  RBC –  WBC-  N - L- M- E- B-  Platelet -  ESR -  -  -  -  -  Na - K- Cl- HCO_2_/CO_2_- |  |
| 3.3 | Therapy  (Give a tick mark if the drug was given to the patient) | 1=Antibiotic (Which one? ………………)  2=Metronidazole  3=Diazepam/Midazolam  4=Magnesium Sulphate  5= Baclofen  6= Dantrolene  7= Barbiturates (Which one?................)  8=Chlorpromazine  9= TIG (Amount?.................)  9=Others………… |  |
| 3.4 | Ventilatory support | Given /couldn’t be given /not needed |  |

**Section D: In-hospital outcome**

| 4.1 | Complications:  (Give a tick mark if the patient has developed one or more of the complications) | 1. Dysautonomia  2. Aspiration pneumonia  3. Hypoxemia (SaO_2_ < 90%)  4. Generalized sepsis  5. Septic shock  6. Bedsore  7. Deep Vein Thrombosis  8. Thrombophlebitis  9. Urinary tract infection  10. Acute renal failure  11. Rhabdomyolysis  12. Others |  |
| --- | --- | --- | --- |
| 4.2 | Outcome | 1=Recovery –  a. with complication  b. without complication  2=Death  3=Referred to ICU  Date of the outcome……………… |  |

**Thank you for your kind participation**
