# Supplementary material for: Factors associated with in-hospital mortality of adult tetanus patients–a multicenter study from Bangladesh
Source: PLoS Negl Trop Dis. 2022 Mar 1;16(3):e0010235. doi: 10.1371/journal.pntd.0010235 (PMC8887756; doi:10.1371/journal.pntd.0010235)
Supplement: S2 Table — (DOCX) [file pntd.0010235.s002.docx]

**S2 Table: Ablett classification^1^ of tetanus severity**

| **Grades** | **Features** |
| --- | --- |
| **Grade 1: Mild** | - Mild to moderate trismus - Generalized spasticity - No respiratory compromise - No spasms - Little or no dysphagia |
| **Grade 2: Moderate** | - Moderate trismus - Marked rigidity - Mild to moderate but short spasms - Moderate respiratory compromise with an increased respiratory rate (>30 breaths per min) - Mild dysphagia |
| **Grade 3: Severe** | - Severe trismus - Generalised spasticity - Reflex prolonged spasms - Increased respiratory rate (>40 breaths per min) - Apnoeic spells - Severe dysphagia - Tachycardia (>120 beats per min) |
| **Grade 4: Very Severe** | - Clinical features of grade 3 tetanus - Violent autonomic disturbances involving the cardiovascular system - Severe hypertension and tachycardia alternating with relative hypotension and bradycardia (either of which might be persistent) |

**Reference**

1. Yen LM, Thwaites CL. Tetanus. The Lancet. 2019;393(10181):1657–1668. doi:10.1016/S0140-6736(18)33131-3. (The authors originally cited it from- Ablett JJL. Analysis and main experiences in 82 patients treated in the Leeds Tetanus Unit. In: Ellis M, ed. Symposium on tetanus in Great Britain. Boston Spa: National Lending Library, 1967: 1–10.)
