# Supplementary material for: Factors associated with in-hospital mortality of adult tetanus patients–a multicenter study from Bangladesh
Source: PLoS Negl Trop Dis. 2022 Mar 1;16(3):e0010235. doi: 10.1371/journal.pntd.0010235 (PMC8887756; doi:10.1371/journal.pntd.0010235)

PLOS Neglected Tropical Diseases

Factors Associated with In-Hospital Mortality of Adult Tetanus Patients– A Multicenter Study from Bangladesh

--Manuscript Draft--

**Manuscript Number:**	PNTD-D-21-01768
**Full Title:**	Factors Associated with In-Hospital Mortality of Adult Tetanus Patients– A Multicenter Study from Bangladesh
**Short Title:**	Factors Associated with Mortality of Adult Tetanus Patients
**Article Type:**	Research Article
**Keywords:**	Tetanus; In-hospital mortality; Fatality; Risk factors
**Abstract:**	Background: Tetanus, a vaccine-preventable disease, is still occurring in the elderly population of low- and middle-income countries with a high case-fatality rate. The objective of the study was to elucidate the factors associated with in-hospital mortality of tetanus in Bangladesh.<br>Methods: This prospective observational study, conducted in two specialized infectious disease hospitals, conveniently selected adult tetanus patients (≥18 years) for inclusion. Data were collected through a preformed structured questionnaire. Kaplan Meier survival analysis and univariate and multivariable Cox regression analysis were carried out to assess factors associated with in-hospital mortality among patients. All analysis was done using Stata (version 16) and SPSS (version 26).<br>Results: A total of 61 confirmed tetanus cases were included, and the overall in-hospital mortality rate was 34.4% (n=21). Patients had an average age of 46.49 ±15.65 years (SD), and the majority were male (96.7%), farmers (57.4%), and came from rural areas (93.4%). Survival analysis revealed that the probability of death was significantly higher among patients having an age of < 40 years, incubation time of ≤12 days, onset time of ≤ 4 days, and having complication(s). However, on multivariable Cox regression analysis, age (adjusted hazard ratio [aHR] 4.03, 95% Confidence Interval [CI] 1.07 – 15.17, p=0.039) and onset time (≤4 days) (aHR 3.33; 95% CI 1.05 – 10.57, p=0.041) came as significant predictors of in-hospital mortality after adjusting for incubation period and complications.<br>Conclusion: Older age and short onset time are the two most important determinants of in-hospital mortality of tetanus patients. Hence, these patients require enhanced emphasis and care.
**Additional Information:**	
**Question**	**Response**
**Financial Disclosure**<br><br>Enter a financial disclosure statement that describes the sources of funding for the work included in this submission. Review the submission guidelines for detailed requirements. View published research articles from *PLOS NTDs* for specific examples.<br><br>This statement is required for submission and **will appear in the published article** if the submission is accepted. Please make sure it is accurate.	The funders had no role in study design, data collection and analysis, decision to publish, or preparation of the manuscript.

Powered by Editorial Manager® and ProduXion Manager® from Aries Systems Corporation

**Unfunded studies**<br><br>Enter: *The author(s) received no specific funding for this work.*<br><br>**Funded studies**<br><br>Enter a statement with the following details:<br>• Initials of the authors who received each award<br>• Grant numbers awarded to each author<br>• The full name of each funder<br>• URL of each funder website<br>• Did the sponsors or funders play any role in the study design, data collection and analysis, decision to publish, or preparation of the manuscript?<br>• **NO** - Include this sentence at the end of your statement: *The funders had no role in study design, data collection and analysis, decision to publish, or preparation of the manuscript.*<br>• **YES** - Specify the role(s) played.<br><br>* typeset	
**Competing Interests**<br><br>Use the instructions below to enter a competing interest statement for this submission. On behalf of all authors, disclose any competing interests that could be perceived to bias this work—acknowledging all financial support and any other relevant financial or non-financial competing interests.<br><br>This statement **will appear in the published article** if the submission is accepted. Please make sure it is accurate. View published research articles from *PLOS NTDs* for specific examples.	The authors have declared that no competing interests exist.

Powered by Editorial Manager® and ProduXion Manager® from Aries Systems Corporation

**NO authors have competing interests**<br><br>Enter: *The authors have declared that no competing interests exist.*<br><br>**Authors with competing interests**<br><br>Enter competing interest details beginning with this statement:<br><br>*I have read the journal's policy and the authors of this manuscript have the following competing interests: [insert competing interests here]*<br><br>* typeset<br><br>This statement is required for submission and **will appear in the published article** if the submission is accepted. Please make sure it is accurate and that any funding sources listed in your Funding Information later in the submission form are also declared in your Financial Disclosure statement.	
**Data Availability**<br><br>Authors are required to make all data underlying the findings described fully available, without restriction, and from the time of publication. PLOS allows rare exceptions to address legal and ethical concerns. See the PLOS Data Policy and FAQ for detailed information.	Yes - all data are fully available without restriction

Powered by Editorial Manager® and ProduXion Manager® from Aries Systems Corporation

A Data Availability Statement describing where the data can be found is required at submission. Your answers to this question constitute the Data Availability Statement and **will be published in the article**, if accepted.<br><br>**Important:** Stating 'data available on request from the author' is not sufficient. If your data are only available upon request, select 'No' for the first question and explain your exceptional situation in the text box.<br><br>Do the authors confirm that all data underlying the findings described in their manuscript are fully available without restriction?	
**Describe where the data may be found in full sentences. If you are copying our sample text, replace any instances of XXX with the appropriate details.**<br><br>• If the data are **held or will be held in a public repository**, include URLs, accession numbers or DOIs. If this information will only be available after acceptance, indicate this by ticking the box below. For example: *All XXX files are available from the XXX database (accession number(s) XXX, XXX.).*<br>• If the data are all contained **within the manuscript and/or Supporting Information files**, enter the following: *All relevant data are within the manuscript and its Supporting Information files.*<br>• If neither of these applies but you are able to provide **details of access elsewhere**, with or without limitations, please do so. For example:<br><br>*Data cannot be shared publicly because*	All relevant data are within the manuscript and its Supporting Information files.

of [XXX]. Data are available from the XXX Institutional Data Access / Ethics Committee (contact via XXX) for researchers who meet the criteria for access to confidential data.<br><br>*The data underlying the results presented in the study are available from (include the name of the third party and contact information or URL).*<br>• This text is appropriate if the data are owned by a third party and authors do not have permission to share the data.<br><br>* typeset	
Additional data availability information:	

Powered by Editorial Manager® and ProduXion Manager® from Aries Systems Corporation

Manuscript
Click here to access/download;Manuscript;Manuscript Tetanus Factors _ PLOS NTD.docx

Title: Factors Associated with In-Hospital Mortality of Adult Tetanus Patients– A Multicenter Study from Bangladesh

Short title: Factors Associated with Mortality of Adult Tetanus Patients

Author' name:

Md. Abdullah Saeed Khan[1], Mohammad Jahid Hasan[2], Md Utba Rashid[3], Soumik Kha Sagar[3], Sanzida Khan[1], Susmita Zaman[2], Sultan Mahamud Sumon[1], Ariful Basher[1], Mohammad Delwer Hossain Hawlader[4], Mohammad Hayatun Nabi[4], Nadira Sultana Kakoly[4]

Authors' Affiliations

1. Infectious Disease Hospital, Mohakhali, Dhaka-1212, Bangladesh.
2. Pi Research Consultancy Center, Dhaka 1211, Bangladesh.
3. Nutrition and Clinical Services Division (NCSD), International Centre for Diarrhoeal Disease Research, Bangladesh (icddr,b), Mohakhali, Dhaka-1212, Bangladesh.
4. Department of Public Health, North South University, Dhaka 1229, Bangladesh.

Corresponding Author:

Md. Abdullah Saeed Khan

MBBS, MPH

Medical Officer

Infectious Disease Hospital,

Mohakhali, Dhaka – 1212, Bangladesh

Phone: +8801823210718

abdullahdmc@gmail.com; abdullah.saeed@northsouth.edu

https://orcid.org/0000-0002-0707-0437

Mobile no: +8801823210718

ABSTRACT

Background: Tetanus, a vaccine-preventable disease, is still occurring in the elderly population of low- and middle-income countries with a high case-fatality rate. The objective of the study was to elucidate the factors associated with in-hospital mortality of tetanus in Bangladesh.

Methods: This prospective observational study, conducted in two specialized infectious disease hospitals, conveniently selected adult tetanus patients (≥18 years) for inclusion. Data were collected through a preformed structured questionnaire. Kaplan Meier survival analysis and univariate and multivariable Cox regression analysis were carried out to assess factors associated with in-hospital mortality among patients. All analysis was done using Stata (version 16) and SPSS (version 26).

Results: A total of 61 confirmed tetanus cases were included, and the overall in-hospital mortality rate was 34.4% (n=21). Patients had an average age of 46.49 ±15.65 years (SD), and the majority were male (96.7%), farmers (57.4%), and came from rural areas (93.4%). Survival analysis revealed that the probability of death was significantly higher among patients having an age of < 40 years, incubation time of ≤12 days, onset time of ≤ 4 days, and having complication(s). However, on multivariable Cox regression analysis, age (adjusted hazard ratio [aHR] 4.03, 95% Confidence Interval [CI] 1.07 – 15.17, p=0.039) and onset time (≤4 days) (aHR 3.33; 95% CI 1.05 – 10.57, p=0.041) came as significant predictors of in-hospital mortality after adjusting for incubation period and complications.

Conclusion: Older age and short onset time are the two most important determinants of in-hospital mortality of tetanus patients. Hence, these patients require enhanced emphasis and care.

Keywords: ***Tetanus; In-hospital mortality; Fatality; Risk factors.***

Synopsis

The incidence of tetanus decreased considerably from the developed countries through mass vaccination programs. However, it continues to be a cause of death in low- and middle-income settings. Tetanus mortality after hospitalization is dependent on many factors. Our study found that more than one-third of the adult (≥18 years) tetanus patients died in the hospitals without intensive care facilities, and patients' older age, short incubation period (time from injury to the appearance of symptom), short onset time (interval between the first symptoms and the first spasm) and development of complications were significantly associated with deaths. Meticulous and individualized management of adult tetanus patients with one or more of the above features are required to increase their survival.

Introduction

Tetanus, an acute and fatal infection, is caused by the neurotoxin-producing bacterium *Clostridium tetani*. It shows a classical clinical picture of muscular rigidity and generalized spasms (1). The spores of *C. tetani* are resilient, long-lasting, and widespread in the environment. It can contaminate wounds, abrasions, and the umbilical stump in neonates(2). Globally nearly one million people contract tetanus annually. It has a high case fatality rate, with an estimated 45.5% (95% CI: 43.7%-47.2%) deaths in African countries(3). However, it might vary based on the availability and accessibility of intensive care units that are well-equipped. Extensive vaccination coverage has led to a decline in the number of new cases of tetanus in developed countries. However, it is still common in low- and middle-income countries. South Asia and Sub-Saharan Africa account for 82% of tetanus cases worldwide(4). In Bangladesh the prevalence is still unknown(5).

Any unvaccinated person has the potential risk of developing tetanus because of the absence of immune protection from natural infection. As childhood and maternal vaccination was started at the end of the twentieth century, tetanus now occurs mainly among older adults in developed countries. On the other hand, neonatal tetanus, which occurs primarily due to inadequate or lack of women's immunization, is frequently found in developing or underdeveloped settings (6). Despite the availability of inexpensive and effective tetanus vaccines, the disease continues to be a health problem in impoverished regions of the world (7).

C tetani spores invade the human body through wounds or minor abrasions under suitable anaerobic conditions. Whenever a wound occurs, appropriate wound care and vaccination could prevent tetanus(8). If infection occurs, patients often die in the hospital due to autonomic failure, cardiovascular dysrhythmia, and complications (9). Proper management can improve survival among patients(10). However, despite appropriate management, many patients fail to survive.

Death in tetanus is dependent on many factors. The poor prognostic factors of in-hospital deaths identified are short incubation period, older age, severe type, generalized variety, dysautonomia, pneumonia, hypoxemia, sepsis, and renal failure(11). Hence, the treatment of cases often requires assisted ventilation. Additionally, passive immunization is usually given following trauma or injury (12). Despite widespread vaccination and advances in management, the mortality in generalized tetanus is still high. Previously, very few studies prospectively explored the factors associated with in-hospital mortality among adult tetanus patients. Moreover, there are few to no studies regarding in-hospital mortality and its associated factors among adult tetanus patients in Bangladesh. Therefore, this research aimed to analyze the factors affecting mortality in hospitalized adult tetanus patients.

Materials and Methods

Study design, population, and settings

This prospective observation was carried out in two specialized infectious disease hospitals in Bangladesh (Infectious Disease Hospital, Mohakhali, Dhaka, and Surya Kanto Hospital, Mymensingh), between December 2020 to August 2021. We approached all adult (≥18 years) hospitalized cases of tetanus inclusion. Patients who were not willing to participate were excluded. A total of 61 clinically diagnosed tetanus patients were conveniently selected for the study.

Study measures

After an extensive review of the published literature, we produced a structured questionnaire for data collection (Supplementary file 1). The questionnaire had four parts: a.) sociodemographic information, b.) information regarding tetanus, c.) comorbidities, investigations, and treatment, d.) in-hospital outcome.

Sociodemographic information

This part queried the patient's age, sex, religion, education, marital status, occupation, monthly family income, and smoking history.

Information regarding tetanus

This part asked about the mode of injury, place of wound, onset time (duration between onset of symptoms and full-blown expression), incubation period, clinical features (including symptoms and signs) at admission, vaccination history (both previous and

postexposure), tetanus types, and investigations. The clinical characteristics comprised of- incubation period (time from injury to the appearance of symptom), time of onset or onset time (interval between the first symptoms and the first spasm), trismus (lockjaw), risus sardonicus (a characteristic, abnormal, sustained spasm of the facial muscles), dysphagia, muscle spams (other skeletal muscles), spasticity, rigidity (overall), abdominal rigidity, opsithotonus (spasm of the muscles causing backward arching of the head, neck, and spine), fever, palpitation, urinary retention and vital signs.

Comorbidities, investigations, and treatment

Patients' comorbidities, including diabetes mellitus, hypertension, chronic obstructive pulmonary disease, chronic renal failure, stroke, and ischemic heart disease, were assessed in this section. Additionally, the information about routine investigations (hematological profile, serum creatinine, serum calcium, and electrolytes) and treatment received during the hospital stay was also collected.

In-hospital outcome

Complications and final outcomes of the patients were listed in this section.

Study procedure

At first, informed written consent was taken from the patients or attendants of unconscious patients at admission for inclusion into this study. Then we recorded patients' socio-economic data, vaccination history, clinical characteristics, and comorbidity data upon inclusion. Patients presenting with lockjaw (trismus) or risus

sardonicus and one or more features from dysphagia, muscle spasms, abdominal rigidity, opisthotonos, and history of the wound were considered to have clinically confirmed tetanus. The presence of an infected wound was considered an essential diagnostic clue in the absence of trismus or risus sardonicus. After the initial assessment, patients were followed up during the hospital stay until discharge with recovery or death. Finally, investigation reports and in-hospital outcome data, including complications were recorded in the questionnaire.

Statistical Analysis

After data entry and curation, we performed descriptive and analytic statistics. Descriptive statistic was expressed as frequency (proportion) for categorical variable and mean ± standard deviation or median (interquartile range) for continuous variable. Univariate analyses were conducted using the chi-square test, independent samples t test, Mann-Whitney U test, and Kaplan Meyer Survival analysis. Univariate and multivariable Cox regression analyses were used for the assessment of significant factors associated with death. Only statistically significant factors ($p<0.05$) at univariate analysis, including age, incubation period, onset time, and presence of complication, were considered for Cox regression. A p-value of ≤ 0.05 was considered significant. Statistical analysis was performed using statistical software Stata (version 16).

Ethical Consideration

The study was approved by the Ethical Review Committee of North South University (2020/OR-NSU/IRB-No.0801). All procedures were conducted following guidelines laid out by the World Medical Association Declaration of Helsinki. Informed written consent was obtained from pa[illegible]ts or attendants of unconscious patients before their inclusion in the study.

Result

The prevalence of in-hospital mortality was 34.4% (21 out of 61) in this study. (**Figure 1).**

Figure 1. In-hospital mortality of tetanus patients

The average age of all patients was 46.49±15.65 years. The mean age of patients who died (52.10 ±12.99 years) was significantly higher than that of recovered patients (43.55 ±16.26 years, p=0.042). Among patients who died, 80.9% were aged ≥ 40 years, and among those who were alive, 55.0% had the same age (p=0.045). The majority patients were male (96.7%), illiterate (46.7%), married (76.7%), farmer (57.4%), had income <7500 BDT (42.6%), and came from rural area (93.4%). The distribution was statistically similar among patients who were alive and who died (**Table 1**).

Table 1. Sociodemographic characteristics of the patients (n=61)

**Variable**	**Total**<br>**n (%)**	**Recovered**<br>**n (%)**	**Died**<br>**n (%)**	**p-value**
**Age (years), mean±SD**	46.49 ±15.65	43.55 ±16.26	52.10 ±12.99	**0.042**
**Age category (years)**				
< 40	22 (36.1)	18 (45.0)	4 (19.1)	**0.045**
≥ 40	39 (63.9)	22 (55.0)	17 (80.9)	
**Sex**				

Male	59 (96.7)	38 (95.0)	21 (100.0)	0.297
Female	2 (3.28)	2 (5.0)	0	
**Education**				
Illiterate	28 (46.7)	18 (45.0)	10 (50.0)	0.986
Primary	11 (27.5)	5 (25.0)	16 (26.7)	
SSC	13 (21.7)	9 (22.5)	4 (20.0)	
HSC and above	3 (5.0)	2 (5.0)	1 (5.0)	
**Marital Status**				
Unmarried	9 (15.0)	6 (15.4)	3 (14.3)	0.967
Married	46 (76.7)	30 (76.9)	16 (76.2)	
Widowed/divorced	5 (8.3)	3 (7.7)	2 (9.5)	
**Occupation**				
Farmer	35 (57.4)	20 (50.0)	15 (71.4)	0.273
Businessman	6 (9.8)	6 (15.0)	0	
Service holder	5 (8.2)	3 (7.5)	2 (9.5)	
Carpenter	4 (6.7)	1 (2.5)	3 (14.3)	
Housewife	2 (3.3)	2 (5.0)	0	
Student	2 (3.3)	1 (2.5)	1 (4.8)	
Electrician	2 (3.3)	2 (5.0)	0	
Retired	2 (3.3)	2 (5.0)	0	
Tailor	1 (1.6)	1 (2.5)	0	
Driver	1 (1.6)	1 (2.5)	0	
Fisherman	1 (2.5)	1 (1.6)	0	
**Income (BDT)**				

<7500	26 (42.6)	18 (45.0)	8 (38.1)	0.382
7501 – 10000	13 (21.3)	9 (22.5)	4 (19.1)	
10001 – 15000	16 (26.2)	11 (27.5)	5 (23.8)	
>15000	6 (9.8)	2 (5.0)	4 (19.0)	
**Residence**				
Rural	57 (93.4)	39 (97.5)	18 (85.7)	0.077
Urban	4 (6.56)	1 (2.5)	3 (14.3)	

p-value determined using independent samples t test and Chi-square test where appropriate; Significant p-values were shown in bold face.

Of all, 8.2% had at least one comorbidity, 6.7% had hypertension, 4.9% had diabetes mellitus, 1.6% had COPD, and 1.6% had ischemic heart disease. Thirty-six percent of patients were current smokers, 20% were past smokers, and 44% never smoked (Table 2).

Table 2. Comorbidity and smoking habit of the patients (n=61)

Variable	Total<br>n (%)	Recovered<br>n (%)	Died<br>n (%)	p-value
**Comorbidity**				
Present (any)	5 (8.2)	2 (5.0)	3 (14.3)	0.209
Absent	56 (91.8)	38 (95.0)	18 (85.7)	
**Diabetes Mellitus**				
Present	3 (4.9)	1 (2.5)	2 (9.5)	0.228
Absent	58 (95.1)	39 (97.5)	19 (90.5)	
**Hypertension**				
Present	4 (6.7)	1 (2.5)	3 (14.3)	0.077
Absent	57 (93.4)	39 (97.5)	18 (85.7)	
**Chronic Obstructive Pulmonary Disease**				
Present	1 (1.6)	0	1 (4.8))	0.164
Absent	60 (98.4)	40 (100.0)	20 (95.2)	
**Ischemic heart disease**				
Yes	1 (1.6)	0	1 (4.8)	0.164
No	60 (98.4)	40 (100.0)	20 (95.2)	
**Smoking habit***				
Current smoker	9 (36.0)	7 (36.8)	2 (33.3)	0.940
Past smoker	5 (20.0)	4 (21.1)	1 (16.7)	
Never smoker	11 (44.0)	8 (42.1)	3 (50.0)	

*After excluding missing cases

p-value determined by Chi-square test

Table 3 presents the clinical characteristics of the patients. Only 6.6% of patients asserted a history of vaccination against tetanus, and 24.6% of patients took postexposure prophylaxis. The main mode of injury was trauma (95.1%), and the majority had their wound in the extremities (91.8%). Most of the patients had the generalized type of tetanus (93.4%) and severe disease (45.9%). These features were statistically similar between alive and dead patients. The overall median onset time was 3 days (interquartile range [IQR]: 2 – 7). It was statistically similar among patients who died (2.5, IQR 2- 4) than those who were alive (4, IQR 2 – 10, p=0.076). The median incubation period was 14 days (IQR 8 – 15) overall. It was also statistically similar between deceased patients (12, IQR 7 – 15) and alive patients (15, IQR 10 – 16, p =0.058). However, when categorized at a cutoff point of 12 days, a statistically significantly higher proportion of dead patients had an incubation period of ≤12 days compared to those alive (p=0.043). Of all patients, the majority had severe trismus (33.9%), mild dysphagia (50%), short spasms (73.5%), and generalized rigidity (93.4%). Spasticity was present in 75.4% of patients, abdominal rigidity in 68.9%, fever in 41%, opisthotonus in 9.8%, and urinary retention in 9.8%. Vital signs were within the normal range. The distribution of the clinical features were statistically similar between patients who was alive and those who died.

Table 3. Clinical characteristics of the patients (n=61)

Variable	Total<br>n (%)	Recovered<br>n (%)	Died<br>n (%)	p-value
**Prior history of vaccination against tetanus**				
Present	4 (6.6)	3 (7.5)	1 (4.8)	0.566
Absent	19 (31.2)	14 (35.0)	5 (23.8)	
Don’t know	38 (62.3)	23 (57.5)	15 (71.4)	
**Postexposure prophylaxis**				
Taken	15 (24.6)	10 (25.0)	5 (23.8)	0.602
Not taken	40 (65.6)	25 (62.5)	15 (71.4)	
Don’t know	6 (9.8)	5 (12.5)	1 (4.8)	
**Mode of injury**				
Post-traumatic	58 (95.1)	37 (92.5)	21 (100.0)	0.209
Postsurgery	3 (4.9)	3 (7.5)	0	
**Place of wound**				
Extremities	56 (91.8)	36 (90.0)	20 (95.2)	0.479
Head/face	5 (8.2)	4 (10.0)	1 (4.8)	
**Tetanus type**				
Generalized	57 (93.4)	37 (92.5)	20 (95.2)	0.681
Localized	4 (6.7)	3 (7.5)	1 (4.8)	

**Time of onset (days), median (IQR)***	3 (2 – 7)	4 (2 – 10)	2.5 (2 – 4)	0.076
> 4 days	20 (36.4)	16 (45.7)	4 (20.0)	0.057
≤ 4 days	35 (63.6)	19 (54.3)	16 (80.0)	
**Incubation period (days), median (IQR)***	14 (8 – 15)	15 (10 – 16)	12 (7 – 15)	0.058
> 12 days	28 (52.8)	21 (63.6)	7 (35.0)	**0.043**
≤ 12 days	25 (47.2)	12 (36.4)	13 (65.0)	
**Tetanus severity**				
Mild	11 (18.0)	8 (20.0)	3 (14.3)	0.871
Moderate	18 (29.5)	12 (30.0)	6 (28.6)	
Severe	28 (45.9)	18 (45.0)	10 (47.6)	
Very severe	4 (6.6)	2 (5.0)	2 (9.5)	
**Trismus***				
Absent	1 (1.7)	1 (2.6)	0	0.677
Mild	8 (13.6)	6 (15.4)	2 (10.0)	
Moderate	30 (50.8)	21 (53.8)	9 (45.0)	
Severe	20 (33.9)	11 (28.2)	9 (45.0)	
**Risus sardonicus**				
Present	5 (8.2)	4 (10.0)	1 (4.8)	0.479
Absent	56 (91.8)	36 (90.0)	20 (95.2)	
**Dysphagia***				

Absent	6 (10.7)	3 (8.6)	3 (14.3)	0.378
Mild	28 (50.0)	20 (57.1)	8 (38.1)	
Severe	22 (39.3)	12 (34.3)	10 (47.6)	
**Spasms***				
Short	25 (73.5)	15 (71.4)	10 (76.9)	0.724
Prolonged	9 (26.5)	6 (28.6)	3 (23.1)	
**Spasticity**				
Present	46 (75.4)	31 (77.5)	15 (71.4)	0.601
Absent	15 (24.6)	9 (22.5)	6 (28.6)	
**Rigidity**				
Localized	4 (6.6)	3 (7.5)	1 (4.8)	0.681
Generalized	57 (93.4)	37 (92.5)	20 (95.2)	
**Abdominal rigidity**				
Present	42 (68.9)	26 (65.0)	16 (76.2)	0.370
Absent	19 (31.2)	14 (35.0)	5 (23.8)	
**Opisthotonus**				
Present	6 (9.8)	6 (15.0)	0	0.062
Absent	55 (90.2)	34 (85.0)	21 (100.0)	
**Fever**				
Present	25 (41.0)	15 (37.5)	10 (47.6)	0.445
Absent	36 (59.0	25 (62.5)	11 (52.4)	
**Palpitation**				

Present	6 (9.8)	4 (10.0)	2 (9.5)	0.953
Absent	55 (90.2)	36 (90.0)	19 (90.5)	
**Urinary retention**				
Present	6 (9.8)	6 (15.0)	0	0.069
Absent	55 (90.2)	34 (85.0)	21 (100.0)	
**Pulse (b/min)**, mean±SD	82.9 ±1.6	80.0 ±1.9	88.2 ±2.3	**0.011**
**Systolic blood pressure (mmHg),** mean±SD	111.4 ±1.9	109.0 ±2.1	115.8 ±3.6	0.087
**Diastolic blood pressure (mmHg),** mean±SD	73.0 ±1.4	73.9 ±1.9	71.5 ±1.9	0.433
**Temperature (F),** mean±SD	97.8 ±0.6	97.2 ±0.9	98.7 ±0.3	0.214
**Respiratory rate** (breaths/min)	20 ±0.6	19.5 ±0.8	20.9 ±0.9	0.256

*Excluding missing values

p-value determined by Mann-Whitney U test, independent samples t test and Chi-square test where appropriate; Significant p-values are shown in bo[illegible]ce.

Hematological profile, random blood sugar, serum creatinine, calcium, and electrolytes were statistically similar between alive and dead patients except for serum sodium

which was significantly higher in patients who died (141.9±1.8 mmol/l) than that of those who were alive (138.0±0.9 mmol/l, p=0.034). However, the average sodium level was within the normal range for both groups of patients (**Table 4**).

Table 4. Investigation profile of participants

Variable	Total Mean ±SD	Recovered Mean ±SD	Died Mean ±SD	p-value
**Hemoglobin (g/dl)**	12.2 ±0.2	12.2 ±0.3	12.1 ±0.5	0.800
**RBC (10^9/mm^3)**	4.44 ±0.2	4.3 ±0.2	4.7 ±0.2	0.394
**WBC (10^3/mm^3)**	9.9 ±0.5	9.4 ±0.6	10.9 ±0.6	0.154
**Neutrophil (%)**	73.1 ±1.4	71.6 ±1.6	76.9 ±2.5	0.091
**Lymphocyte (%)**	20.8 ±1.3	22.4 ±1.6	17.1 ±2.2	0.065
**Platelet (10^6/mm^3)**	237.6 ±12.8	240.9 ±16.5	229.5 ±19.1	0.694
**ESR (mm)**	22.8 ±1.7	22.7 ±2.2	23.4 ±2.6	0.853
**RBS (mmol/l)**	7.1 ±0.5	7.3 ±0.7	6.8 ±0.3	0.604
**Serum calcium (mg/dl)**	7.9 ±0.03	7.9 ±0.05	7.9 ±0.03	0.765
**Serum creatinine (mg/dl)**	1.2 ±0.1	1.2 ±0.1	1.2 ±0.1	0.948
**Serum electrolyte (mmol/l)**				
**Sodium**	139.3 ±0.9	138.0 ±0.9	141.9 ±1.8	0.034
**Potassium**	4.4 ±0.7	3.7 ±0.1	5.6 ±1.8	0.178
**Chloride**	107.5 ±1.02	106.2 ±1.1	109.8 ±1.9	0.095

RBC: Red blood cell; WBC: White blood cell

p-value determined by independent samples t test; Significant p-values were shown in bold face

Out of 61 patients, 26 (42.6%) developed at least one complication, and this proportion was significantly higher in dead patients (61.9%) compared to that of alive patients (32.5%, p=0.027). The most common complication was hypoxemia (31.1%) followed by aspiration pneumonia (19.6%), bedsore (13.1%), dysautonomia (6.5%), DVT (4.9%), UTI (3.28%), sepsis (1.6%), thrombophlebitis (1.6%), and wound infection (1.6%). The individual distribution of the complications was statistically similar between the alive and dead patient groups (**Table 5**).

Table 5. List of complications among patients (n=61)

**Variable**	**Total**<br>**n (%)**	**Recovered**<br>**n (%)**	**Died**<br>**n (%)**	**p-value**
**Any complication**				
Present	26 (42.6)	13 (32.5)	13 (61.9)	**0.027**
Absent	35 (57.4)	27 (67.5)	8 (38.1)	
**Hypoxemia**				
Present	19 (31.1)	6 (15.0)	13 (61.9)	0.164
Absent	42 (68.9)	34 (85.0)	8 (38.1)	

**Aspiration Pneumonia**				
Present	12 (19.6)	5 (12.5)	7 (33.3)	0.05
Absent	57 (93.4)	39 (97.5)	18 (85.7)	
**Bedsore**				
Present	8 (13.1)	7 (17.5)	1 (4.8)	0.161
Absent	53 (86.9)	33 (82.5)	20 (95.2)	
**Dysautonomia**				
Present	4 (6.5)	2 (5.0)	2 (9.5)	0.498
Absent	57 (93.4)	38 (95.0)	19 (90.5)	
**DVT**				
Present	3 (4.9)	1 (2.5)	2 (9.5)	0.228
Absent	58 (95.1)	39 (97.5)	19 (90.5)	
**UTI**				
Present	2 (3.28)	2 (5.0)	0	0.297
Absent	59 (96.7)	38 (95.0)	21 (100.0)	
**Sepsis**				
Present	1 (1.6)	1 (2.5)	0	0.465
Absent	60 (98.4)	39 (97.5)	21 (100.0)	
**Thrombophlebitis**				
Present	1 (1.6)	0	1 (4.8)	0.164
Absent	60 (98.4)	40 (100.0)	20 (95.2)	
**Wound infection**				

Present	1 (1.6)	1 (2.5)	0	0.465
Absent	60 (98.4)	39 (97.5)	21 (100.0)	

p-value determined by Chi-square test; Significant p-values are shown in bold face; DVT: Deep Vein Thrombosis; UTI: Urinary Tract Infection.

The median duration of hospital stay of the patients was 11 days (IQR: 6 – 21 days) (**Figure 2**). Recovered patients had a significantly longer duration of stay (median 17 days; IQR: 10 – 23 days) than those who died (median 5 days; IQR: 2 – 8 days) ($p<0.001$).

Figure 2. Boxplots showing the duration of hospital stay among tetanus patients categorized by outcome

Kaplan-Meier survival analysis showed that the probability of survival among tetanus patients at 40 days after admission was 0.6, and it was statistically significantly better among patients with an age of < 40 years, incubation time of ≤12 days, onset time of ≤ 4 days and no complications (**Figure 3**).

Figure 3. Kaplan-Meier survival curves showing the probability of overall survival and survival across different groups of tetanus patients

Only factors that were significant (p<0.05) in Kaplan-Meier survival analysis were considered for univariate and multivariable Cox regression models to determine their associations with the in-hospital mortality of tetanus patients (Table 6). After adjusting for incubation period and complications, only age (≥40 years) and onset time (≤4 days) were found to be significant predictors of tetanus case-fatality in the hospital. Tetanus patients aged ≥40 years were 4.03 times (95% CI 1.07 – 15.17, p=0.039) more likely to die due to tetanus than those aged<40 years. Patients with an onset time of ≤4 days were significantly more likely (aHR 3.33; 95%CI 1.05 – 10.57, p=0.041) to die in the hospital than those with a higher onset time.

Table 6. Univariate and multivariable Cox regression analysis for factors associated with in-hospital mortality among tetanus patients

**Factors**	**Reference Category**	**Crude HR (95%CI)**	**p-value**	**Adjusted HR (95%CI)**	**p-value**
Age group (≥40 years)	<40 years	2.98 (0.99 – 9.02)	0.053	4.03 (1.07 – 15.17)	**0.039**
Incubation period (≤12 days)	>12 days	2.57 (0.96 – 6.85)	0.059	2.49 (0.90 – 6.90)	0.078
Onset time (≤4 days)	>4 days	2.94 (0.97 – 8.97)	0.058	3.33 (1.05 – 10.57)	**0.041**

Complication (Present)	Absent	2.79 (1.06 – 7.34)	**0.038**	2.09 (0.72 – 6.07)	0.175

HR: Hazard ratio; Significant p-values are shown in boldface.

Discussion

Tetanus is a vaccine-preventable disease that is still continuing to infect people, particularly, in low-~~income~~ and middle-income countries. As the spore of the causative organism *C. tetani* remains widespread in the environment, its eradication is impossible. On the other hand, life-long immunity against tetanus requires three booster doses of vaccine during the adolescent years (13). Currently, only women of childbearing age are targeted through booster vaccination programs, which have substantially reduced maternal and neonatal tetanus in many countries. However, it remains a problem for adults in South Asia and Sub-Saharan Africa. The Global Disease Burden (GBD) studies suggested that, as of 2017, approximately 82% of all tetanus cases in the world were comprised of patients from these two regions, along with 77% of the total 38,000 tetanus deaths (4). Hence, it was pertinent to study the factors associated with mortality among patients who are already infected. This study is one of the few attempts in Bangladesh to explore the factors associated with in-hospital mortality of adult tetanus patients.

We found that nearly 34.4% of patients died in the hospital. This is higher than two previous studies from Bangladesh, where authors reported 22.5%(14) and 28.6%(5) deaths. However, the observed death rates show regional variations. For instance,

Tanon et al. reported a death rate of 30% in the Ivory Coast (15), and Marulappa et al. found it to be 42.2% in India(16). The case-fatality rates appear to be considerably lower where patients were provided ventilatory support. This can be seen in China (17) and Nepal (18), where only 5.9% and 7.5% of deaths were reported, respectively. However, despite giving mechanical ventilation, the death rate was 43.1% in Tanzania(19), a lower-middle-income country, which raises the importance of overall management facilities as well as other factors associated with deaths among tetanus patients.

We noted that patients who died had a significantly higher mean age than those who were alive, and patients with age ≥ 40 years had an increased chance of death in the hospital. This finding is supported by previous observations in Bangladesh(5), Tanzania(19), and India(16). Similar to ours, Chalya et al. (19) noted that patients aged ≥40 years were significantly more likely to die when adjusted for other factors, such as incubation period, onset time, presence of complications, prior immunization, and tetanus severity.

The male population constituted the maximum number of participants in our study as well as other studies reporting on tetanus cases(7,14–19). This probably reflects the outcome of immunization strategies for women of childbearing age in all countries. The WHO recommends three booster dosages of vaccine at ages: 12 – 23 months, 4 – 7 years, and 9 – 15 years for achieving life-long immunity for all (13). Although many countries have programs for childhood immunization, expanding those to include

booster dosages remains challenging. Hence, the vulnerable male population should be identified and vaccinated to protect them from tetanus ~~as a starter~~.

One important finding of our study is that more than half of the patients were farmers and lived in rural areas. Similarly, many previous studies(16,19–22) observed a high proportion of farmers among tetanus patients. As *C. tetani* spores remain in the environment, people working in the fields, often with sharp cutting instruments, have a higher chance of being exposed to the bacteria due to accidental punctures or lacerated wounds. Farmers or agriculturists in low- and middle-income countries often work barefooted without adequate personal protective measures in the fields(23). Therefore, they could be treated as a high-risk group to prioritize primary and booster immunization wherever appropriate.

We found that only 6.6% of patients could remember taking tetanus vaccine in the past and only one-quarter of patients took postexposure prophylaxis. Similar observations were reported by Khakheli et al. (22) and Tosun et al. (6). Patients tend to forget the previous history of vaccination, and their attendants are even less likely to know about their vaccination history, which explains the reports. However, a low frequency of post-exposure prophylaxis indicates a lack of knowledge, awareness, and practice regarding tetanus vaccination after injury, particularly minor injuries, among the general population. Health authorities should work to address this issue where tetanus is not uncommon.

Although an onset time (duration between onset of symptoms to full-blown presentation) of 48 hours (2 days) and incubation of period of 7 days is used as a cutoff

point of determining the increased risk of fatality in the majority of studies exploring prognostic factors of tetanus(2), we selected a higher cutoff point for both because the median onset time and incubation period were high among our study participants. Our analysis revealed that an onset time of $\leq$ 4 days and an incubation period of $\leq$12 days significantly increased the chance of fatality of tetanus patients in the hospital. However, after adjustment for age and the presence of complications, only onset time ($\leq$4 days) remained a significant determinant of in-hospital mortality. Previously, Amare et al. (11) reported a univariate association of <3 days onset time with mortality, which became statistically nonsignificant after multivariate logistic adjustments. Similarly, Krishnan et al. (12) and Chalya et al. (19) didn't find any association of onset time ($\leq$48 hours) with the death of tetanus patients. The incubation period of <7 days was found to be associated with a significantly increased risk of mortality in ~~several~~ previous reports(19,22). In contrast, some other reports did not find such an association(7,11). However, very few studies have considered a higher incubation period cutoff point for analysis, making it difficult to comment on the findings. Additionally, differences in sample size and consideration of other factors during multivariate adjustment as well as the type of analysis used, often influence the outcome. Hence, the findings should be read in the context of a particular study.

The clinical presentation of tetanus shows variability in previous studies. The most common symptoms among our patients included lockjaw (trismus), dysphagia, and spasticity like previous observations in Bangladesh(5,14). We did not find any noticeable differences in clinical features between alive and dead tetanus patients. Neither did we find any remarkable differences in routine investigations of dead and

alive patients. Serum sodium levels were slightly but statistically significantly higher in deceased patients than that of recovered ones. However, both groups had their values within the normal limit. Therefore, the difference discovered could be a random finding that needs to be evaluated through further large sample studies.

Unlike Chalya et al. (19), we did not find an increased risk of mortality in patients with severe tetanus. But our finding is concordant with that of Amare et al. (11). However, it might depend on the scales used for severity grading among patients. The Ablett classification scheme used in our study is dependent on the presence of spasms and autonomic disturbance, which evolve throughout the course of the disease (2). Hence it is considered to be less suitable for prognostic stratification. Nevertheless, similar to other studies(22), we noted that nearly 50% of patients presented with severe and very severe disease.

In the present study, only 8.2% of participants had one or more comorbidities from diabetes, hypertension, COPD, and ischemic heart disease. However, it was not associated with a higher risk of mortality. Rather, the development of complication(s) was associated with a significantly higher risk of death in the hospital, which is concordant with findings from Marulappa et al. (16), Tanon et al. (15), Krishnan et al. (12), and Bankole et al. (20). Hypoxemia and aspiration pneumonia were frequent complications that we encountered in our patients. Hypoxemia is the consequence of laryngeal spasms, respiratory distress, and respiratory failure that might occur in tetanus patients(12). Unlike Amare et al. (11), who reported the occurrence of dysautonomia in 91.7% of deceased tetanus patients, we found the problem in only 9.5% of patients who

died. This could be explained by the lack of cardiac monitoring facilities and intensive care units in our centers, the presence of which would have allowed close monitoring and precise detection of autonomic imbalance in these patients. Moreover, life-saving ventilatory supports for patients were not possible either because of the lack of facilities.

Limitations

Our study had several limitations. The sample size was small.. The impact of ventilatory support on mortality could not be evaluated due to a lack of facilities. Further follow-up of patients after discharge from the hospital was also not possible. However, this was one of the few attempts to assess the clinical-epidemiological profile and factors associated with in-hospital mortality among tetanus patients in Bangladesh. Our findings would undoubtedly spark interest in further studies and inform policymakers regarding the limitations in the management of tetanus that need to be addressed.

Recommendations

Considering the findings of this study, we have the following recommendations-

1. Adult tetanus patients with a higher age should be given special care during management.
2. A shorter onset time of tetanus must warrant careful assessment of disease severity for meticulous treatment.
3. A booster vaccination program should be started prioritizing vulnerable male population to prevent tetanus incidence in the country.

4. Further large-scale countrywide studies on tetanus patients should be carried out to explore management strategies and reduce case-fatality among tetanus patients.

Conclusion

Although expanded programs on immunization and maternal and neonatal tetanus elimination programs have been successful in the considerable reduction of tetanus in neonates, children, women of childbearing age, and men at their early adulthood, older adult men are still vulnerable to tetanus in Bangladesh. As most of the cases come from rural areas where farming and manual work are the principal modes of earning, they could be considered a priority group for a booster vaccination program. On the other hand, tetanus patients with higher age and shorter onset time needs special care during management as they have a higher risk of death.

Authors Declaration

Declaration of Competing Interest: The authors declare that there are no conflicts of interest.

Authors Contribution:

The conception of the study was generated by MASK. A detailed outline and design of the study was prepared by MASK, MJH, SZ, SMS, AB, MDHH MHN and NSK. Data collection, data acquisition and associated works were performed by MASK, SK, MUR, and SKS. Data analysis was carried out by MASK. The first draft of the manuscript was prepared by MASK. MASK, MJH, MUR, SKS, SK, SZ, SMS, AB, MDHH, MHN, NSK, reviewed the draft and approved the final version.

Data available: Available upon reasonable request from the corresponding authors.

Funding: No funding was received for this study.

Acknowledgement: Authors acknowledge the contribution of the patients and their guardians for their kind consent to participate in the study.

Reference:

1. Bennett JE, Dolin R, Blaser MJ. Infectious Disease Essentials. Philadelphia: Elsevier; 2017.

2. Yen LM, Thwaites CL. Tetanus. Lancet. 2019;393(10181):1657–68.

3. Woldeamanuel YW, Andemeskel AT, Kyei K, Woldeamanuel MW, Woldeamanuel W. Case fatality of adult tetanus in Africa: Systematic review and meta-analysis. J Neurol Sci. 2016;368:292–9.

4. Yen L, Thwaites C, Behrens H, Ochmann S, Danoaite B, Roser M. Tetanus [Internet]. OurWorldInData.org. 2019. p. 1657–68. Available from: https://ourworldindata.org/tetanus

5. Hasnain MG, Maruf S, Nath P, Anuwarul A, Ahmed MNU, Chowdhury IH, et al. Managing severe tetanus without ventilation support in a resource-limited setting in Bangladesh. Am J Trop Med Hyg. 2018;99(5):1234–8.

6. Tosun S, Batirel A, Oluk AI, Aksoy F, Puca E, Bénézit F, et al. Tetanus in adults: results of the multicenter ID-IRI study. Eur J Clin Microbiol Infect Dis. 2017;36(8):1455–62.

7. Saltoglu N, Tasova Y, Midikli D, Burgut R, Dündar IH. Prognostic factors affecting deaths from adult tetanus. Clin Microbiol Infect. 2004;10(3):229–33.

8. Erdem H, Inan A, Altindis S, Carevic B, Askarian M, Cottle L, et al. Surveillance, control and management of infections in intensive care units in Southern Europe, Turkey and Iran - A prospective multicenter point prevalence

study. J Infect. 2014;68(2):131–40.

9. Lisboa T, Ho Y-L, Henriques Filho GT, Brauner JS, Valiatti JLDS, Verdeal JC, et al. Guidelines for the management of accidental tetanus in adult patients. Rev Bras Ter intensiva. 2011;23(4):394–409.

10. Ajose FOA, Odusanya OO. Survival from adult tetanus in Lagos, Nigeria. Trop Doct. 2009;39(1):39–40.

11. Amare A, Melkamu Y, Mekonnen D. Tetanus in adults: Clinical presentation, treatment and predictors of mortality in a tertiary hospital in Ethiopia. J Neurol Sci. 2012;317(1–2):62–5.

12. Khrisnan L, Anam, Panigoro R. Factors Affecting Mortality in Adult Tetanus Patients. Althea Med J. 2015;2(2):157–62.

13. World Health Organization. Protecting all against tetanus. Vol. 92. 2017. 1–6 p.

14. Feroz A, Rahman M. A Ten-year Retrospective Study of Tetanus at a Teaching Hospital in Bangladesh. J Bangladesh Coll Physicians Surg. 2007;25(2).

15. Tanon AK, Doumbia A, Coffie PA. Current Prognostic Factors of Tetanus in Abidjan: 2005-2014. J Microbiol Infect Dis. 2017;7(June):125–31.

16. Marulappa VG, Manjunath R, Mahesh N, Maligegowda L. A ten year retrospective study on adult Tetanus at the epidemic disease (ED) hospital, Mysore in Southern India: A review of 512 cases. J Clin Diagnostic Res. 2012;6(8):1377–80.

17. Fan Z, Zhao Y, Wang S, Zhang F, Zhuang C. Clinical features and outcomes of tetanus: A retrospective study. Infect Drug Resist. 2019;12:1289–93.

18. Shah B, Subedi M, Bartaula B, Ghimire A. Retrospective chart- review of Tetanus cases admitted in a tertiary care hospital. J Adv Intern Med. 2020;9(2):69–72.

19. Chalya PL, Mabula JB, Dass RM, Mbelenge N, Mshana SE, Gilyoma JM. Ten-year experiences with Tetanus at a Tertiary hospital in Northwestern Tanzania: A retrospective review of 102 cases. World J Emerg Surg. 2011;6(1):2–9.

20. Bankole IA, Danesi MA, Ojo OO, Okubadejo NU, Ojini FI. Characteristics and outcome of tetanus in adolescent and adult patients admitted to the Lagos University Teaching Hospital between 2000 and 2009. J Neurol Sci. 2012;323(1–2):201–4.

21. Ramachandra L, Shobha K, Kannan PA. A Retrospective Clinical Study of Factors Affecting Tetanus. internet J Microbiol. 2008;7(1):1–6.

22. Khakheli MS, Khuhro BA, Jamali AH. Tetanus: Still a killer in adults. Anaesthesia, Pain Intensive Care. 2013;17(2):149–53.

23. Öncü SS, Önde M, Öncü SS, Ergin F, Öztürk B. Tetanus seroepidemiology and factors influencing immunity status among farmers of advanced age. Health Policy (New York). 2011;100(2–3):305–9.

Click here to access/download;Figure;Figure 1.png

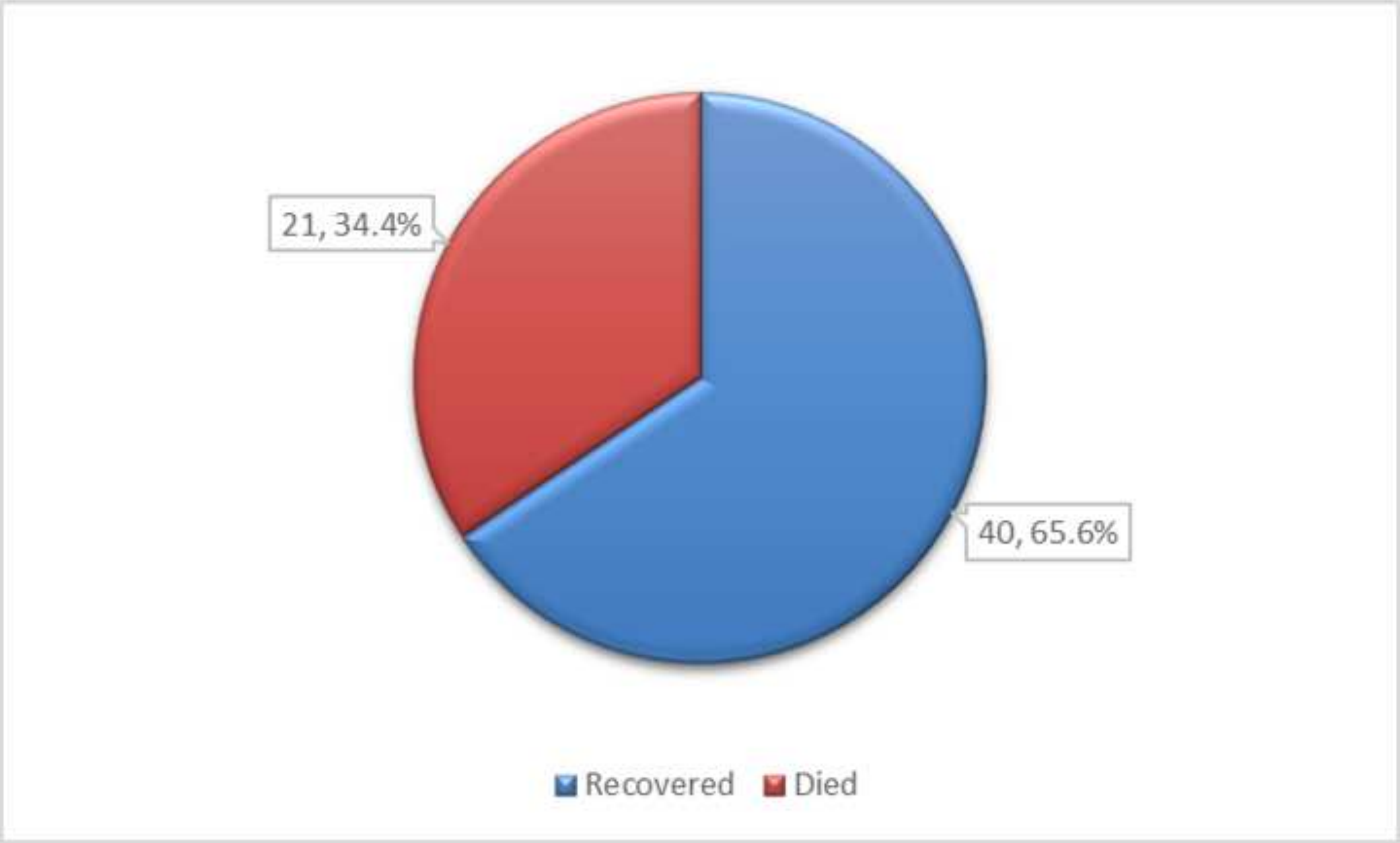

Click here to access/download;Figure;Figure 2.tiff

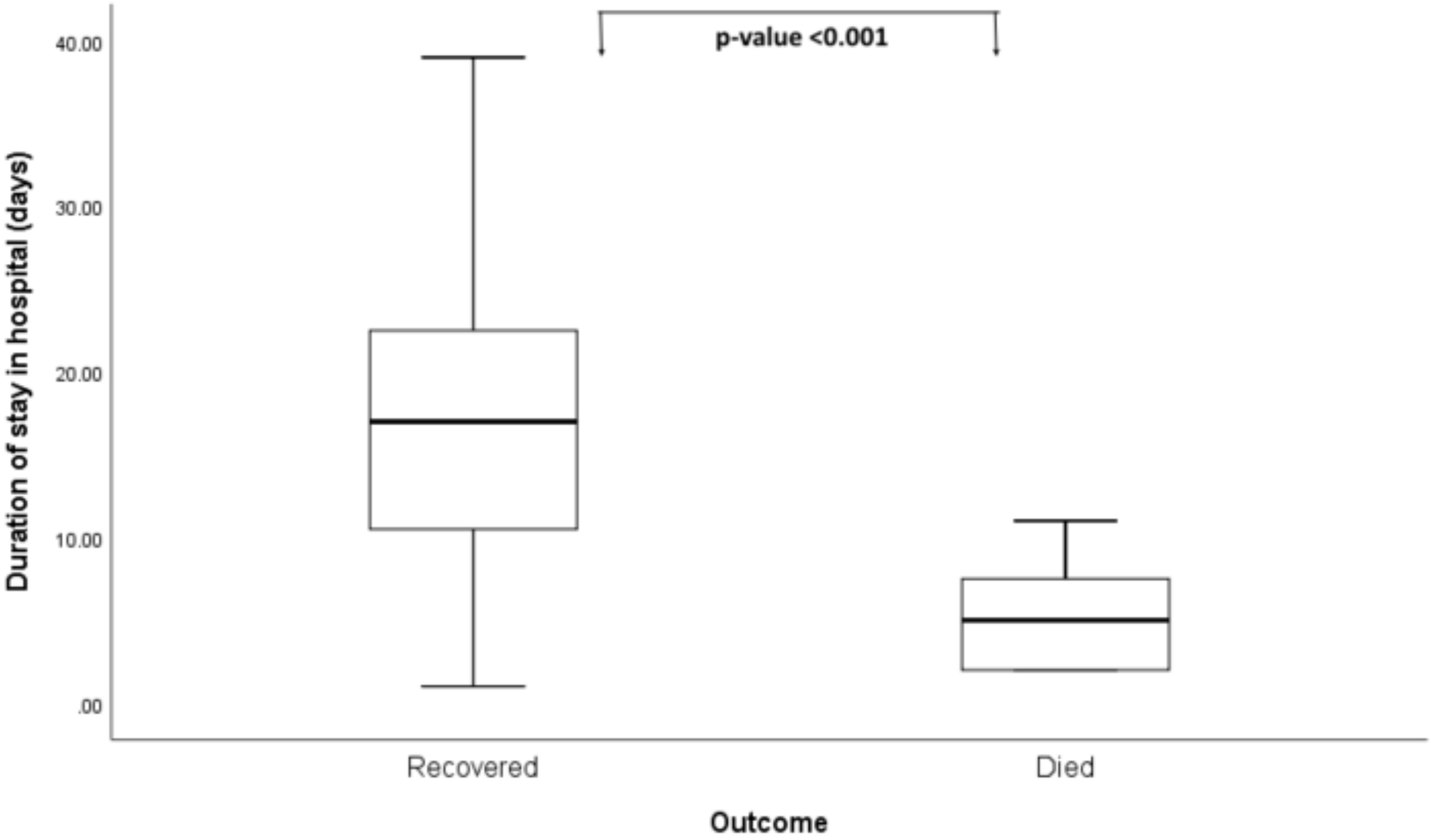

Click here to access/download;Figure;Figure 3.png

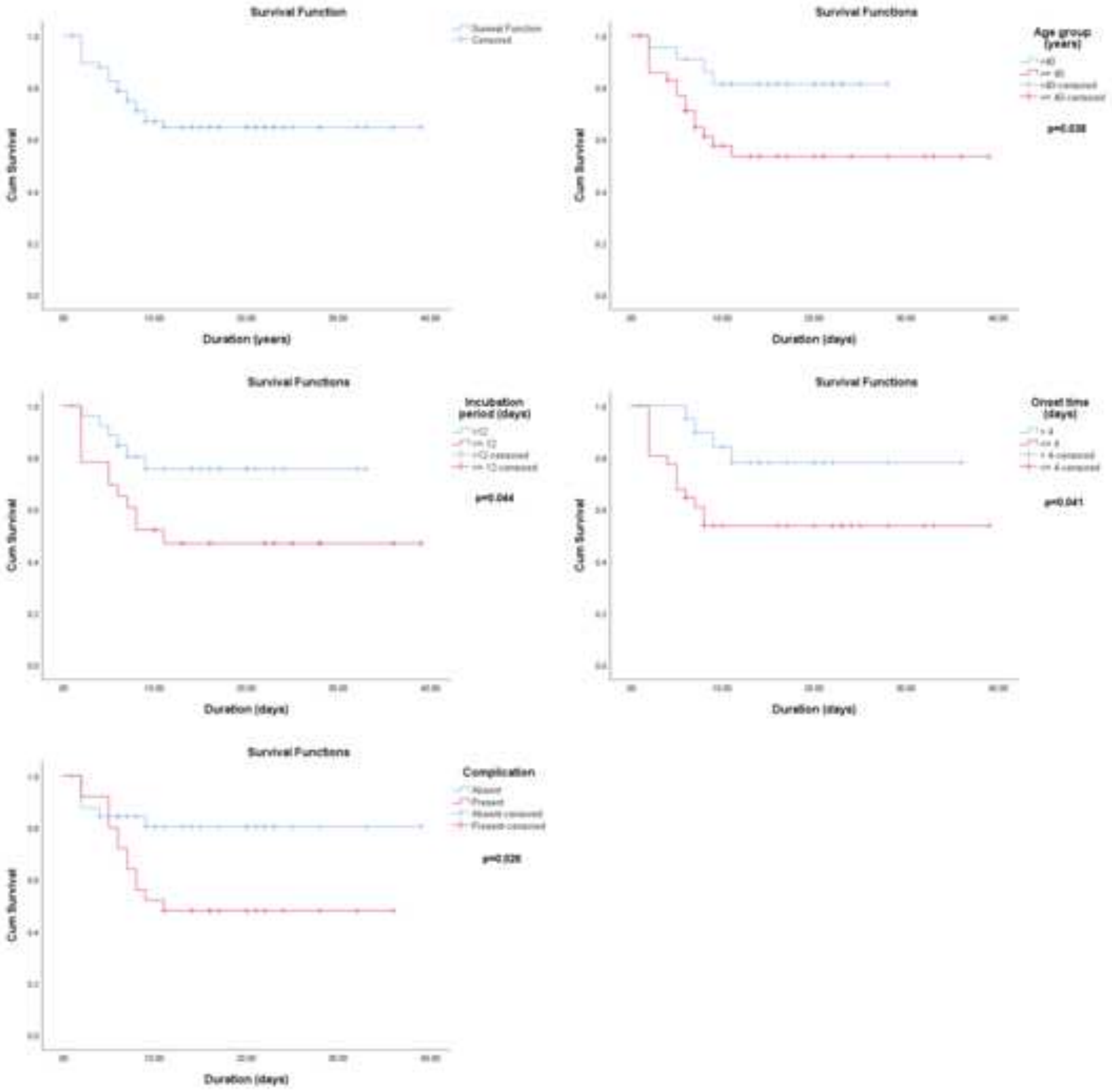

Supplement: Attachment — Submitted filename: PNTD-D-21-01768_EO.pdf [file pntd.0010235.s004.pdf]
