# Supplementary material for: Factors associated with in-hospital mortality of adult tetanus patients–a multicenter study from Bangladesh
Source: PLoS Negl Trop Dis. 2022 Mar 1;16(3):e0010235. doi: 10.1371/journal.pntd.0010235 (PMC8887756; doi:10.1371/journal.pntd.0010235)
Supplement: Attachment — Submitted filename: Review of PNTD.doc [file pntd.0010235.s003.doc]

Review suggestion on

“Factors Associated with In-Hospital Mortality of Adult Tetanus Patients– A Multicenter Study from Bangladesh “

Manuscript Number: PNTD-D-21-01768

General Comments:

The objective of this study was to understand the factors associated with in-hospital mortality of adult Tetanus patients in two centers of Bangladesh between December 2020 to August 2021. To this end the researchers performed an observational study using a sample of 61 confirmed tetanus cases. Though Tetanus is a rare case in developed countries, still cause of death from tetanus is a matter of concern in developing and under developed countries. Being a study in Bangladesh where Tetanus is still on the table of Health policy makers, this study is very time demanding and output of this study will guide health department of the country to rethink the hospital facilities to reshape their services.

However, the study seems good to go on but it has some minor issues which should be solved earlier.

Here are some suggestions and corrections to be corrected before publishing.

Specific Comments:

Comment 1:

- Line 48: Survival analysis revealed that the probability of death was significantly higher among patients having an age of <40. But according Table 1, this should be ≥  40

Comment 2:

- Line 48: Incubation time ≤ 12 days, onset time  ≤ 4 days is determined on which reference.

Comment 3:

- Line 181: ‘The’ instead of ‘he’

Comment 4:

- In Figure 3, Graph 1, Duration is expressed as Years, which should be Days according your description.
