# Supplementary material for: Factors associated with in-hospital mortality of adult tetanus patients–a multicenter study from Bangladesh
Source: PLoS Negl Trop Dis. 2022 Mar 1;16(3):e0010235. doi: 10.1371/journal.pntd.0010235 (PMC8887756; doi:10.1371/journal.pntd.0010235)
Supplement: Attachment — Submitted filename: Tetanus NTD comments.docx [file pntd.0010235.s005.docx]

Tetanus NTD comments

Abstract:

Line 44: what is the definition of confirmed tetanus?

Line 48: in abstract age < 40 years has been mentioned as predictors of death but in the text in other places mentioned >40 years. (Line 54)

Materials:

Line 116- incomplete sentence

Facilities available in each hospital including availability of intensive care and assisted respiratory support may be briefly mentioned.

Line 183: Legend of the figure is incomplete (n=? )

Line 117: how many cases were excluded

Line 186: 80.9% were aged >40 years but inconsistent what has been mentioned in the abstract

Line 189: income 7500 BDT, is it per month? What is the conversion of BDT into USD?

Table 1: need correction: widow, income eper month,

Line 195-196- not clear

Definition of post exposure prophylaxis?

What is meant by mode of injury and sub types?

Definition of ‘localized’ and ‘generalized’ tetanus may be given

Time of onset- needs clarification

Definition of ‘severity score’ and ‘trismus severity score’ may be given in the method’

Any photo of ‘trismus’- if available with consent may be provided

Facilities of intensive care and assisted rei hospitals managing the cases of tetanusspiration be made available in Bangladesh.

Who evaluated the patients in the two hospitals and how uniformity of assessment was maintained?

Discussion:

Instead of many unnecessary points, focused discussion on the factors causing high death may be critically discussed.
