# Supplementary material for: Factors associated with in-hospital mortality of adult tetanus patients–a multicenter study from Bangladesh
Source: PLoS Negl Trop Dis. 2022 Mar 1;16(3):e0010235. doi: 10.1371/journal.pntd.0010235 (PMC8887756; doi:10.1371/journal.pntd.0010235)
Supplement: Attachment — Submitted filename: Response to reviewers.docx [file pntd.0010235.s006.docx]

Dear Editor,

First, we want to express our heartiest thanks and gratitude to the reviewers and editors for their nice review and constructive comments. Their comments had been instrumental in improving our manuscript. A point-by-point response to the reviewers’ comments are appended below.

The responses are arranged as follows. For each reviewer, first a response is provided against reviewer’s responses to questions (if there is any). This is followed by a response to their comments.

**Reviewer 1**

**Reviewer's Responses to Questions**

**Authors’ comments:** As the respected reviewer didn’t have any comments in this section no response is provided.

**Comments by reviewer 1**

Review suggestion on

“Factors Associated with In-Hospital Mortality of Adult Tetanus Patients– A Multicenter Study from Bangladesh “

Manuscript Number: PNTD-D-21-01768

**General Comments:**

The objective of this study was to understand the factors associated with in-hospital mortality of adult Tetanus patients in two centers of Bangladesh between December 2020 to August 2021. To this end the researchers performed an observational study using a sample of 61 confirmed tetanus cases. Though Tetanus is a rare case in developed countries, still cause of death from tetanus is a matter of concern in developing and under developed countries. Being a study in Bangladesh where Tetanus is still on the table of Health policy makers, this study is very time demanding and output of this study will guide health department of the country to rethink the hospital facilities to reshape their services.

However, the study seems good to go on but it has some minor issues which should be solved earlier.

**Authors’ comments:** We are very thankful to the reviewer for these nice comments. We hope our study will highlight the current situation associated with tetanus management in both national and international community.

Here are some suggestions and corrections to be corrected before publishing.

**Specific Comments:**

**Comment 1:**

- Line 48: Survival analysis revealed that the probability of death was significantly higher among patients having an age of <40. But according Table 1, this should be ≥ 40
- **Authors’ comments:** Thank you for pointing this out. We have corrected the age. Please check line 48.

**Comment 2:**

- Line 48: Incubation time ≤ 12 days, onset time ≤ 4 days is determined on which reference.
- **Authors’ comments:** Thank you for the query. Most of the previous studies has taken a cut-off point of 7 days for incubation period and 48 hours (2 days) for onset time. However, in our study we first statistically tested the incubation period and onset time of our patients by their outcome using Mann-Whitney U test (as the data was skewed). We found that a cut-off point of 12 days for incubation period and 4 for onset time creates the best point of difference across our outcome variable (i.e., alive or dead). Hence, this cut-off point was taken not based on any previous reference but on the basis of our statistical findings.

**Comment 3:**

- Line 181: ‘The’ instead of ‘he’
- **Authors’ comments:** Thank you for the small correction. The word was corrected. Please check line 196.

**Comment 4:**

- In Figure 3, Graph 1, Duration is expressed as Years, which should be Days according your description.
- **Authors’ comments:** Thank you very much for pointing this out. We have corrected the word. We have also added improved and more clear version of the figures, particularly figure 3.

**Reviewer 2**

**Reviewer's Responses to Questions**

Key Review Criteria Required for Acceptance?
As you describe the new analyses required for acceptance, please consider the following:

**Methods**
-Are the objectives of the study clearly articulated with a clear testable hypothesis stated?
-Is the study design appropriate to address the stated objectives?
-Is the population clearly described and appropriate for the hypothesis being tested?
-Is the sample size sufficient to ensure adequate power to address the hypothesis being tested?
-Were correct statistical analysis used to support conclusions?
-Are there concerns about ethical or regulatory requirements being met?

Reviewer #2: The objectives of the study are clearly articulated. The study did not require testing any hypothesis.
The study design was appropriate to address the objectives stated.
The population studied was clearly described and appropriate for the objectives
The sample size is good for the study
The Statistical analysis used were correct to support the conclusions
The ethical requirement will require some modification as the authors claimed that patients with tetanus gave written informed consent. This calls for questioning as a patient with spasm may be unable to give written consent, however the patient may be able to give verbal consent with their caregiver going ahead to sign any document that needs to be signed.

**Authors’ comments:** We are grateful to the reviewer for the nice and constructive comments. We have corrected the ethical review section accordingly and changed it as follows- “…Informed written consent was obtained from patients’ caregivers if the patients were unconscious or unable to write. However, in the latter case a verbal consent was taken from the patient first.” (Please check line 190 to 192).

--------------------

**Results**
-Does the analysis presented match the analysis plan?
-Are the results clearly and completely presented?
-Are the figures (Tables, Images) of sufficient quality for clarity?


Reviewer #2: The analysis presented matched the analysis plan
The results were clearly presented except for the Kaplan Meier Survival Analysis that needs to be reviewed as presently presented by the authors. Table 1 requires some corrections which have been highlighted.
The images and figures are clear to a good extent except for figure 3 which is not so clear.

**Authors’ comments:** Thank you very much for the comments and thank you again for pointing out discrepancy between figure 3 and the description in result section.

We made necessary corrections in the table 1. We have improved the figure 3 to enhance clarity and corrected the corresponding description in results of Kaplan Meier Survival Analysis (please see line 276, 277). The section now reads as follows –

“Kaplan-Meier survival analysis showed that the probability of survival among tetanus patients at 40 days after admission was 0.6, and it was statistically significantly better among patients with an age of < 40 years, incubation time of >12 days, onset time of >4 days, and no complications”.

--------------------

**Conclusions**
-Are the conclusions supported by the data presented?
-Are the limitations of analysis clearly described?
-Do the authors discuss how these data can be helpful to advance our understanding of the topic under study?
-Is public health relevance addressed?

Reviewer #2: The conclusions are supported by the data presented
The limitations of the study were fairly well described
The authors did discuss the relevance of the study viz-a-viz tetanus elimination.
Tetanus is a disease of public Health relevance and this was highlighted in the manuscript.

**Authors’ comments:** Thank you very much for the comments.

--------------------

**Editorial and Data Presentation Modifications?**
Use  this section for editorial suggestions as well as relatively minor modifications of existing data that would enhance clarity. If the only modifications needed are minor and/or editorial, you may wish to recommend “Minor Revision” or “Accept”.

Reviewer #2: The authors have tried. However, the manuscript has lots of errors of syntax and semantics which requires a lot of work.

**Authors’ comments:** Thank you very much for your comments and concerns. Due to lack of funding, we could not use a professional language editing service. However, we have made corrections according to author’s suggestions in the manuscript and thoroughly checked the manuscript using Grammarly Premium application. We believe this has improved the writing.

--------------------

**Summary and General Comments**
Use this section to provide overall comments, discuss strengths/weaknesses of the study, novelty, significance, general execution and scholarship. You may also include additional comments for the author, including concerns about dual publication, research ethics, or publication ethics. If requesting major revision, please articulate the new experiments that are needed.


Reviewer #2: Thanks for asking me to review this manuscript. The topic is of public health relevance. It highlighted the need to focus on adult farmers who are high risk as far as tetanus is concerned. Some concerns have been highlighted as sticky notes in the manuscript and some suggestions made.

**Authors’ comments:** We are very grateful to the reviewer for this nice review and constructive comments. We have tried our best to address the points made in the manuscript which are described below.

**Reviewer’s comments on the manuscript as sticky notes:**

**Line 78:** Please reference this sentence.

**Authors’ comments:** We are very grateful to the reviewer for this comment. As per suggestion of the reviewer while reviewing our reference we noted a mistake in the prevalence of tetanus as described in our text.

It was described as follows- “Globally nearly one million people contracted tetanus annually.”

However, we have now corrected it and changed the description and added the relevant reference in this way- “Globally approximately one hundred thousand people contracted tetanus in 2017(3).” (Please check line 77,78).

**Line 88:** This sentence needs to be referenced please.

**Authors’ comments:** Again, thanks for pointing this out. We have corrected the description and added a relevant reference.

Previously it was written in this way- “As childhood and maternal vaccination was started at the end of the twentieth century, tetanus now occurs mainly among older adults in developed countries.”

We have changed it to write – “As childhood and maternal vaccination, started at the end of the twentieth century, has rapidly reduced the incidence of tetanus throughout the world, it continues to be a substantial problem among older adults in many countries(2).” (Please check line 86- 89).

**Line 113:** prospective observational study

**Authors’ comments:** We have corrected the description accordingly. (Please check line 114).

**Line 114:** The authors will do well by giving an idea of the total number of adult patients managed for tetanus during the study period.

**Authors’ comments:** We added additional description as follows. (Please check line 120– 123).

“A total of 78 patients were admitted during the study period. However, 16 patients didn’t match the age criteria (i.e., they were <18 years) and one patient didn’t give consent. Finally, a total of 61 clinically diagnosed tetanus patients were conveniently selected for the study.”

**Line 113:** prospective observational study

**Authors’ comments:** We have corrected the description accordingly. (Please check line 114).

**Line 128:** site of the wound

**Authors’ comments:** We have replaced ‘place of wound’ with ‘site of the wound’ (Please check line 133).

**Line 128:** were

**Authors’ comments:** Corrected accordingly (Please check line 157).

**Line 157:** with recovery or death as the final outcome.

**Authors’ comments:** Corrected accordingly (Please check line 172).

**Line 165:** Meier

**Authors’ comments:** Corrected accordingly (Please check line 180).

**Line 176:** How did patients with tetanus able to give written informed consent? I will advise the researchers to review this. The caregivers can help the patient to write/ sign the consent form after the patient has given his or her assent.

**Authors’ comments:** Thank you very for bringing this to our attention. We have corrected the description as follows-

“Informed written consent was obtained from patients’ caregivers if the patients were unconscious or unable to write. However, in the latter case a verbal consent was taken from the patient first.” (Please check line 190 – 192).

**Line 181:** The

**Authors’ comments:** Corrected accordingly (Please check line 196).

**Line 189:** Can the dollar equivalent of this be written in bracket by the authors?

**Authors’ comments:** We have added the US dollar equivalents in the description as well in the table 1. (please check line 204).

**Table 1:**

Row 8 (Gender)- Write the value to one decimal place like others.

Row 9 (Education)- All the percentages in the three columns need to be revisited. Some add up to more than 100% while the middle column is less than 100%.

Row 18 (Occupation)- The percentages under this variable also need to be reviewed as the first and second columns are more than 100%.

Row 29 - How come 1 in this first and middle columns have different percentages? This should be corrected.

**Authors’ comments:** Thank you very much for showing us the discrepancy. We checked and found that some of the data was mistakenly placed in different columns. We have checked and corrected all the proportions and frequencies. Also, we have round all the data at one decimal place. (Check table 1).

**Line 193:** The face should be removed. Significant values are written in bold is fine.

**Authors’ comments:** Corrected accordingly. (Please check line no 209).

**Line 226:** bold. Not boldface please.

**Authors’ comments:** Corrected accordingly. (Please check line no 242).

**Line 238:** As above.

**Authors’ comments:** Corrected accordingly. (Please check line no 254).

**Table 5:** Row (UTI): Please use one decimal place like other figures.

**Authors’ comments:** Corrected accordingly. (Please check table 5).

**Line 262:** This seems contradictory to the earlier results that showed that the chances of survival are better with incubation time of > 12 days and onset time of greater than or equal to 4 days. The authors need to review this section of the result for clarity. I think the authors need to rewrite this section to reflect their findings.

**Authors’ comments:** Thank you very much for pointing this out. We have changed the description accordingly. (Please check line 276,277).

“Kaplan-Meier survival analysis showed that the probability of survival among tetanus patients at 40 days after admission was 0.6, and it was statistically significantly better among patients with an age of < 40 years, incubation time of >12 days, onset time of >4 days, and no complications”.

**Line 282:** strikethrough text

**Authors’ comments:** Corrected accordingly. (Please check line 298).

**Line 320:** strikethrough text

**Authors’ comments:** Corrected accordingly. (Please check line 336).

**Reviewer 3**

**Reviewer's Responses to Questions**

Key Review Criteria Required for Acceptance?
As you describe the new analyses required for acceptance, please consider the following:

**Methods**
-Are the objectives of the study clearly articulated with a clear testable hypothesis stated?
-Is the study design appropriate to address the stated objectives?
-Is the population clearly described and appropriate for the hypothesis being tested?
-Is the sample size sufficient to ensure adequate power to address the hypothesis being tested?
-Were correct statistical analysis used to support conclusions?
-Are there concerns about ethical or regulatory requirements being met?

Reviewer #3: Objectives are clear; study design is relatively appropriate; description of the population needs some clarity- example who are excluded with reasons to be mentioned; No concern about ethical aspects.
Who evaluated the patients in the two hospitals and how uniformity in assessment was maintained?

**Authors’ comments:** We are delighted to have such nice reviews from the reviewer. We are also thankful for the constructive comments. In accordance with the suggestions of the reviewer we have described the number of excluded patients with reasons for exclusion. (Please check line 119 – 123). The description is as follows-
“Patients who were not willing to participate were excluded. A total of 78 patients were admitted during the study period. However, 16 patients didn’t match the age criteria (i.e., they were <18 years) and one patient didn’t give consent.”

We have described the ways evaluation patients in two hospitals and of maintaing uniformity of assessment in the study procedure subsection of methods section. (Please check 158 – 163). The section is described as follows-
“The data collection was done by the principal investigator (PI) (MASK) and three co-investigators (UBR, SKS, and SK) of the research. All of them are physicians directly involved in the management and research of tetanus patients in the study centers. To maintain uniformity of data collection all the data collectors were trained on the questionnaire before the study started.”

--------------------

**Results**
-Does the analysis presented match the analysis plan?
-Are the results clearly and completely presented?
-Are the figures (Tables, Images) of sufficient quality for clarity?

Reviewer #3: Table 1: need correction- widow; income per month
Lines 195-196- not clear
Definition of post exposure prophylaxis?
What is meant by mode of injury and sub-types?
Definition of 'localized' and generalized' tetanus may be given
Time of onset- needs clarification
Definition of 'severity score' and 'trismus severity score' may be given in the method.
Any photo of 'trismus' if available with consent may be provided

**Authors’ comments:** Thank you very much for the suggestion. We believe this has substantially improved the methods and results section. We have made the necessary correction in table 1.

Line 195- 196 were changed to increase clarity. It now reads “Of all patients, 6.7% had hypertension, 4.9% had diabetes mellitus, 1.6% had COPD, and 1.6% had ischemic heart disease. Total 8.2% of patients had at least one comorbidity.” (Please check line 211 – 213).

Definition of post-exposure prophylaxis, mode of injury, subtype, localized and generalized tetanus, time of onset were added in the study measures subsection of methods section. We used Albett’s classification of tetanus for severity grading. It was references in the study measures section. A supplementary table was added to include the classification criteria for reference. Definition of severity score and trismus severity scores were also added in study measures subsection. (Please check line no line 142 – 148).

We are extremely unfortunate and sorry, that we don’t have any photo of trismus from our patients taken with permission. Therefore, this could not be provided.

--------------------

**Conclusions**
-Are the conclusions supported by the data presented?
-Are the limitations of analysis clearly described?
-Do the authors discuss how these data can be helpful to advance our understanding of the topic under study?
-Is public health relevance addressed?

Reviewer #3: Limitations of the study: facilities of intensive care assisted respiration in hospitals managing the cases of tetanus may be made available in Bangladesh
Some part of data needs clarity
Public relevance addressed well

**Authors’ comments:** Thank you very much for the suggestion. We have addressed issue in our limitation and tried our best to make the data as clear as possible as per review.
--------------------

**Editorial and Data Presentation Modifications?**
Use this section for editorial suggestions as well as relatively minor modifications of existing data that would enhance clarity. If the only modifications needed are minor and/or editorial, you may wish to recommend “Minor Revision” or “Accept”.

Reviewer #3: Minor Revision

**Authors’ comments:** Thank you for the nice comments and review.

--------------------

Summary and General Comments
Use this section to provide overall comments, discuss strengths/weaknesses of the study, novelty, significance, general execution and scholarship. You may also include additional comments for the author, including concerns about dual publication, research ethics, or publication ethics. If requesting major revision, please articulate the new experiments that are needed.


Reviewer #3: (No Response)

**Authors’ comments:** Thank you again for the nice comments and review.

**Comments by reviewer 3**

Tetanus NTD comments

**Abstract:**

**Line 44:** what is the definition of confirmed tetanus?

**Authors’ comments:** Thank you for the question. We intended to mean clinically confirmed tetanus. However, as the word ‘confirmed’ is creating ambiguity we have removed it from the abstract. (Please check line 44).

**Line 48**: in abstract age < 40 years has been mentioned as predictors of death but in the text in other places mentioned >40 years. (Line 54)

**Authors’ comments:** Thank you for pointing this out. It was a mistake in the abstract. We have corrected the cut-off point in the abstract. (Please check line 48).

**Materials:**

**Line 116**- incomplete sentence

Facilities available in each hospital including availability of intensive care and assisted respiratory support may be briefly mentioned.

**Authors’ comments:** Thank you for the suggestion. We have added a line describing the facilities and lack of intensive care support in the centers studied (Please check line 116-118). The description now reads as follows-
“This prospective observational was carried out in two specialized infectious disease hospitals in Bangladesh (Infectious Disease Hospital, Mohakhali, Dhaka, and Surya Kanto Hospital, Mymensingh), between December 2020 to August 2021. Both of these hospitals have dedicated units (wards) for tetanus patients including all management facilities except the provision of intensive care and assisted ventilation.”

**Line 117**: how many cases were excluded

**Authors’ comments:** Thank you for the suggestion. We have added a description of the excluded cases as follows. (Please check line 120-121.)

**“**A total of 78 patients were admitted during the study period. However, 16 patients didn’t match the age criteria (i.e., they were <18 years) and one patient didn’t give consent.”

**Line 183:** Legend of the figure is incomplete (n=? )

**Authors’ comments:** Thank you for pointing this out. We have added the number of patients in the legend. (Please check line 199.)

**Line 186:** 80.9% were aged >40 years but inconsistent what has been mentioned in the abstract

**Authors’ comments:** Thank you for the point. I think there was a misunderstanding from the sentence due to inadequate description. We have changed the lines as follows to increase clarity. In fact 80.9% were aged ≥ 40 among the patients who died, not among total.

**“**Overall, 63.9% were aged ≥40 years. However, among patients who died, 80.9% were aged ≥ 40 years, and among those who were alive, 55.0% had the same age (p=0.045)”. (Please check line 202, 203).

**Line 189:** income 7500 BDT, is it per month? What is the conversion of BDT into USD?

**Authors’ comments:** Thank you for the point. It is per month. We have added the USD in brackets in description as well as in the table. (Please check table 1 and line 205).

**Table 1:** need correction: widow, income eper month,

**Authors’ comments:** Thank you for the point. Corrected accordingly.

Line 195-196- not clear

**Authors’ comments:** Thank you again. As was responded earlier line 195- 196 were changed to increase clarity. It now reads “Of all patients, 6.7% had hypertension, 4.9% had diabetes mellitus, 1.6% had COPD, and 1.6% had ischemic heart disease. Total 8.2% of patients had at least one comorbidity.” (Please check line 211 – 213).

Definition of post exposure prophylaxis?

What is meant by mode of injury and sub types?

Definition of ‘localized’ and ‘generalized’ tetanus may be given

Time of onset- needs clarification

Definition of ‘severity score’ and ‘trismus severity score’ may be given in the method’

Any photo of ‘trismus’- if available with consent may be provided

Facilities of intensive care and assisted rei hospitals managing the cases of tetanusspiration be made available in Bangladesh.

Who evaluated the patients in the two hospitals and how uniformity of assessment was maintained?

**Authors’ comments:** We have made the necessary corrections as described earlier under the response to question section. However, for your convenience we are copying the responses here.

Definition of post-exposure prophylaxis, mode of injury, subtype, localized and generalized tetanus, time of onset were added in the study measures subsection of methods section. We used Albett’s classification of tetanus for severity grading. It was references in the study measures section. A supplementary table was added to include the classification criteria for reference. Definition of severity score and trismus severity scores were also added in study measures subsection. (Please check line no line 142 – 148).

We are extremely unfortunate and sorry, that we don’t have any photo of trismus from our patients taken with permission. Therefore, this could not be provided.

We have described the ways evaluation patients in two hospitals and of maintaing uniformity of assessment in the study procedure subsection of methods section. (Please check 158 – 163). The section is described as follows-
“The data collection was done by the principal investigator (PI) (MASK) and three co-investigators (UBR, SKS, and SK) of the research. All of them are physicians directly involved in the management and research of tetanus patients in the study centers. To maintain uniformity of data collection all the data collectors were trained on the questionnaire before the study started.”

**Discussion:**

Instead of many unnecessary points, focused discussion on the factors causing high death may be critically discussed.

**Authors’ comments:** Thank you for the valuable suggestion. We have added discussion on the factors associated with death. (please check line 408 to 420). The description is now as follows.
“On multivariable regression analysis, after adjusting for incubation period and presence of complications, age ≥ 40 years and onset time of ≤4 days were found to be significant predictors of in-hospital deaths of patients. Aging is associated with a reduction in antibody levels in individuals previously immunized against tetanus (24,25). Moreover, aging itself shows a declines of the immune system (26), and a rise in the number of chronic conditions (27), leading to a lower healing capacity. All of these factors might combinedly influence the outcome of tetanus in older patients. A short onset time implies a quick evolution of the disease after initiation of symptoms leading to increased severity(2). Hence, aggressive management strategies are required for patients with ≥40 years of age and short onset time. Moreover, assisted ventilation facilities are crucial for critically ill tetanus patients. The high proportion of death observed in this study could be attributed to the lack of intensive care facilities in these centers.”
